# Cdrom Archive: A Gateway to Study Camel Phenotypes

**DOI:** 10.3389/fgene.2019.00048

**Published:** 2019-02-05

**Authors:** Hasan Alhaddad, Bader H. Alhajeri

**Affiliations:** Department of Biological Sciences, Kuwait University, Kuwait City, Kuwait

**Keywords:** camel biobank, camel breed, camel ear, coat color, hair length, hair texture, Mezayen, tail length

## Abstract

Camels are livestock that exhibit unique morphological, biochemical, and behavioral traits, which arose by natural and artificial selection. Investigating the molecular basis of camel traits has been limited by: (1) the absence of a comprehensive record of morphological trait variation (e.g., diseases) and the associated mode of inheritance, (2) the lack of extended pedigrees of specific trait(s), and (3) the long reproductive cycle of the camel, which makes the cost of establishing and maintaining a breeding colony (i.e., monitoring crosses) prohibitively high. Overcoming these challenges requires (1) detailed documentation of phenotypes/genetic diseases and their likely mode of inheritance (and collection of related DNA samples), (2) conducting association studies to identify phenotypes/genetic diseases causing genetic variants (instead of classical linkage analysis, which requires extended pedigrees), and (3) validating likely causative variants by screening a large number of camel samples from different populations. We attempt to address these issues by establishing a systematic way of collecting camel DNA samples, and associated phenotypic information, which we call the “Cdrom Archive.” Here, we outline the process of building this archive to introduce it to other camel researchers (as an example). Additionally, we discuss the use of this archive to study the phenotypic traits of Arabian Peninsula camel breeds (the “Mezayen” camels). Using the Cdrom Archive, we report variable phenotypic traits related to the coat (color, length, and texture), ear and tail lengths, along with other morphological measurements.

## Introduction

Dromedary camels (*Camelus dromedarius* Linnaeus, 1758) are exceptional livestock animals because of their natural adaptations to hot sandy desert environments (Schmidt-Nielsen, 1959; Abu-seida et al., 2012; Eshra and Badawy, 2014) and their artificially selected traits (Farah, 1993; Khalaf, 1999; Kadim et al., 2008; Teague, 2009; Kagunyu et al., 2013).

Despite the seemingly large variation in physiological, biochemical, morphological, and behavioral traits, the camel has received little attention with regard to the documentation of these traits, insofar as their hereditary status and their molecular basis (Burger, 2016). Using various genetic resources (Al-Swailem et al., 2010; Wu et al., 2014; Fitak et al., 2016), few studies have recently started to investigate the genetic basis of camel phenotypic and behavioral traits (Holl et al., 2017; Almathen et al., 2018; Ramadan et al., 2018); mostly using the candidate gene(s) sequencing approach (Zhu and Zhao, 2007).

For example, sequencing the *KIT* (Tyrosine kinase receptor) gene revealed the variants associated with the white-spotting phenotype of piebald (painted) camels (Holl et al., 2017; Volpato et al., 2017). The candidacy of this gene was established based on findings in other animals. The *KIT* gene has been identified or implicated to be related to white color or white-spotting in alpacas (Jackling et al., 2014), cows (Fontanesi et al., 2010), yaks (Zhang et al., 2014), pigs (Cho et al., 2011), goats (Nazari-Ghadikolaei et al., 2018), horses (Hauswirth et al., 2013), donkeys (Haase et al., 2015), cats (David et al., 2014), dogs (Wong et al., 2012), mice (Geissler et al., 1988), and rabbits (Fontanesi et al., 2014). Thus, applying a candidate gene approach to camels requires the presence of the phenotype in other mammals and a manageable number of candidate genes to be sequenced.

Beyond the candidate gene approach (which requires the existence of a similar phenotype in a related mammal), genetic investigations in camels also includes classical linkage analysis (Ott et al., 2015), genome-wide association (Hirschhorn and Daly, 2005), or whole-genome sequencing approaches (Petersen et al., 2017). All these approaches provide an opportunity to study camel-specific characteristics, and for many cases, narrow down the number of candidate genes to investigate. However, several challenges hinder the implementation of these approaches in camels. These challenges include: (1) the limited camel genetic resources (i.e., no high-density SNP array or genome-wide STR panel), (2) the lack of multigenerational pedigrees to conduct linkage analyses, (3) the difficulty of obtaining a pedigree when most camel breeders rely on mental documentation of their crosses (Köhler-Rollefson, 1993), (4) the late breeding age (∼4 years) and the long gestation (∼12 months) and weaning (∼9 months) periods of camels (which prevents any attempt to start a large scale breeding experiment) (Ali et al., 2018), (5) the absence of a detailed record of camel traits or genetic diseases and their likely mode of inheritance (i.e., dominant, recessive, etc.) and heritability, and (6) the lack of camel breed registry or recorded information, especially for desired traits (i.e., milk volume, meat quality, coat color, racing performance, etc.).

All the aforementioned genetic approaches to study camel phenotypes, as well as validation of phenotype-genotype association, require a large number of carefully phenotyped individuals of known ancestry. This necessity justifies the assembly of a camel DNA biobank, which is implemented in other livestock animals (Groeneveld et al., 2016; Blackburn, 2018). Accordingly, we established such a biobank, which we refer to as the *C. dromedarius* Archive (“Cdrom Archive”) that consists of biological specimens (DNA source) accompanied by detailed specimen-associated information, such as age, sex, breed/type, pedigree, location, and a comprehensive documentation of morphological phenotypes in the form of photographs.

In this review, we present our methodology of collecting and organizing each camel sample in the archive. We also use the current samples of the Cdrom Archive to characterize six camel breeds from the Arabian Peninsula (Majaheem, Sofor, Shaele, Homor, Shageh, and Waddeh), with an emphasis on the variation in the coat (i.e., color, length, and texture), ear morphology (i.e., shape and length), and tail length.

## Building and Using the Cdrom Archive

### Data Collection and Organization

**FIGURE 1:**
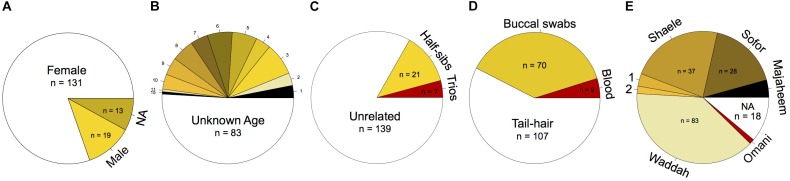
Cdrom Archive sample-specific information. General counts based on **(A)** sex, **(B)** age, **(C)** pedigree information, **(D)** biological specimen type, and **(E)** breed affiliation (1: Homor and 2: Shageh).

Sample-specific information of the Cdrom Archive is collected and organized in a unified format using the *SamplEase* application (Alhaddad and Alhajeri, 2018). While we propose to collect and organize our camel specimens using the aforementioned sample collection application, data can also be included in the Cdrom Archive manually. The archive is currently comprised of 163 samples that were collected during 2015 (February–April), 2016 (October–December), and 2017 (March–April) (Figure 1A and Supplementary Table S4). We plan to continue to add more samples (and associated phenotypic data) to the Cdrom Archive in the future. Our long-term plan is to make the archive available in a database on the web, which will continuously be updated with new specimens, as they are collected. The current Cdrom Archive specimen’s information is listed in Supplementary Table S4, both photographs and biological material associated with each specimen is available upon request.

### Sex Information

Females (*n* = 131) currently represent the majority of the samples of the Cdrom Archive. The discrepancy in the number of female to male (*n* = 19) samples is a consequence of each breeder keeping only one or two reproductively active males in their stock at each time (Ali et al., 2018) (Figure 1A).

### Age Information

**FIGURE 2:**
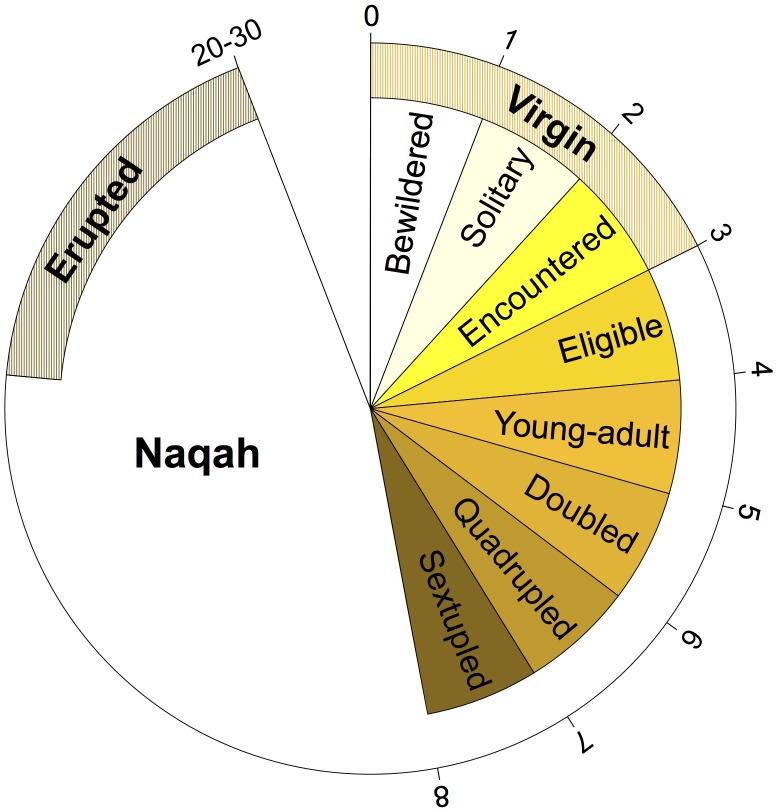
Terminology of Mezayen camel age classes and their approximate age in years. Each age class represents a phase in the growth of a female camel (male terminology is similar) and does not depend on specific number of months/years. Outer numbers are approximate number of years for the completion of each age stage. For details regarding the terms and their meaning refer to Supplementary Table 1.

Samples from camels of various ages were collected. However, the majority of the samples thus far were of unknown age (*n* = 83) (Figure 1B). This is in part due to the lack of a written record of the breeding stock, and the reliance on teeth appearance and camel behavior to determine age. Most camel breeders in the Arabian Peninsula do not keep track of the specific age of each of their camels (i.e., number of years), but rather label their age class generally based on their behavior, reproductive maturity, and teeth development (Figure 2 and Supplementary Table S1). It is thus necessary to use age categories such as juvenile, subadult, and adult instead of years. It is always possible to deduce the age category of each camel sampled in the Cdrom Archive by referring to the associated photographs (see below).

### Pedigree Information

Pedigree information of Cdrom samples is mostly incomplete—it currently contains only seven trios (parents and an offspring) and 21 half-siblings (siblings sharing a single parent) (Figure 1C). It is difficult to obtain pedigreed camel samples because breeders in the Arabian Peninsula (1) rely mostly on a mental record of their breeding programs (Köhler-Rollefson, 1993), (2) assign the same name to multiple camels (which increases the likelihood of pedigree mistakes), (3) constantly exchange/sell camels with other breeders, and (4) use reproductively superior bull camels for breeding (bulls are neither owned by the breeder nor present at the time of sample collection). It is thus easier to locate and collect trios, siblings, half-siblings, or small pedigrees than find a multigenerational pedigree.

### Biological Specimens

The biological specimens of the Cdrom Archive presently come from whole-blood, buccal swabs, and tail-hair (Figure 1D). We found that the most appropriate camel DNA source for the Cdrom Archive is tail-hair follicles—this is based on its ease of collection, transport, and storage, and because it provided adequate DNA quantities for genetic analyses 30 tail-hail follicles ≈6 μg (Alhaddad et al., 2019). The quantity of DNA, obtained from hair follicles, is thus expected to be successfully used in each of PCR, STR and SNP genotyping, targeted sequencing, and whole-genome sequencing.

In the process of establishing the Cdrom Archive, we arrived at the following recommendations to safely collect tail-hair samples (intended as a DNA source). To avoid startling the camel, it should be approached slowly from the front, and then it is advisable to pet the animal to allow it to relax, before moving toward the tail to collect the DNA sample. It was easier to collect tail-hair samples from females, since they tend to be more relaxed, probably since they are used to being milked by the breeders. Unlike horses that kick posteriorly, camels kick sideways, and thus, it is advisable when collecting tail-hair samples to stand behind the camel, and not to its side. To collect hair in an optimal manner, a small bundle of long tail-hairs near the base of the tail can be wrapped around the index finger and plucked upward. It is recommended to bind the hair bundle using tape and discard excess hair away from the roots (tips) (since it does not contain any DNA), before being stored in a labeled envelope.

### Geographic Distribution

**FIGURE 3:**
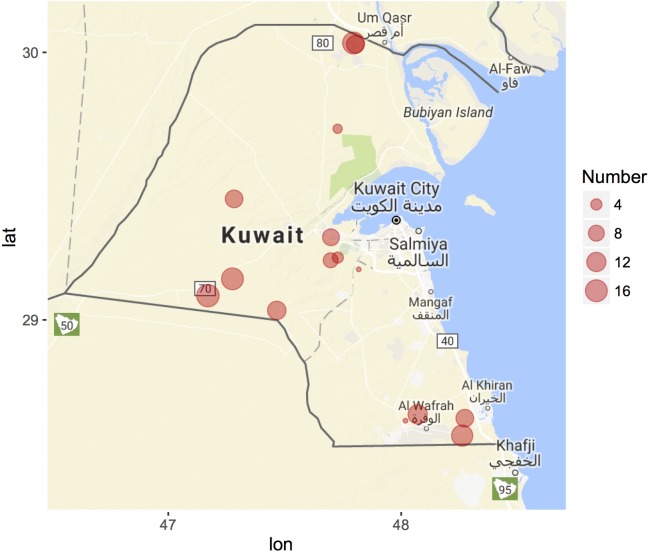
Geographic distribution of Cdrom Archive samples from Kuwait. Samples were collected during 2015 (February–April), 2016 (October–December), and 2017 (March–April). Circle size corresponds to the number of camel samples from each breeder (location). Nine samples were collected from King Faisal University, Saudi Arabia (not shown). Map generated using the *ggmap* R package.

GIS coordinates are automatically assigned to each collected specimen in the Cdrom Archive using *SamplEase* (Alhaddad and Alhajeri, 2018). Most of our samples so far were collected from Kuwait (15 locations) and only nine samples come from Saudi Arabia (all from Alhasa) Figure 3 – map generated using *ggmap* R package (Kahle and Wickham, 2013; R Development Core Team, 2018). We acknowledge that camel herds are generally maintained in an open environment, rather than in a closed farm, and that camel breeders change their location several times to prevent disease due to accumulation of fecal material and to void depleting grazing grounds. Nonetheless, GPS coordinates can be used to accurately reference each sample to its location of collection—this data may allow for the construction of a camel locality heat map, that would be helpful for national census surveys of camel populations, along with disease management and prevention plans e.g., managing the Middle East respiratory syndrome-MERS (Omrani et al., 2015).

### Photographs

*SamplEase* allows for the collection of an unlimited number of photographs for each sampled camel, which are all linked to the basic information for each camel sample. The majority of the sampled camels in the Cdrom Archive have been photographed multiple times—these photographs allow us to subsequently characterize the morphological features of each sampled camel.

### Sampled Breeds in the Cdrom Archive

Most of our samples presently come from Kuwait and consist of camel breeds common in the Arabian Peninsula. The breeds currently in the Cdrom Archive are Majaheem, Sofor, Shaele, Homor, Shageh, Waddah, and Omani (Figure 1E). Many alternative spellings for these breeds exist in the literature; for consistency, we have adopted the spellings used by Porter et al. (2016).

Studying the molecular basis of any trait is more achievable in a breed rather than in a random bred population (Karlsson and Lindblad-Toh, 2008). This is due to the genetic similarity between individuals within a breed compared to an admixed population. The genetic similarity between members of a breed reduces the variable sites to be investigated and enables better localization of phenotype-associated genes. However, the concept of a breed is a subject of historic (Lloyd-Jones, 1915) and ongoing debates (Food Agriculture Organization of the United Nations, 2013), and applying this concept to dromedary camels is even more debatable and harder to implement (Köhler-Rollefson, 1993; Wardeh, 2004; Dioli, 2016). Animal breeds are generally defined based on characteristics agreed upon by breeders that are implemented using documented breed standards, which requires an animal registry, and a governing breed association (Food Agriculture Organization of the United Nations, 2013). The camel breeding community suffers from the lack any breeders’ associations or organizations – such communities often set breed defining criteria and features for other animals. The closest to a camel breed registry or a governing body is the Camel Race Federation in the United Arab Emirates (Khalaf, 1999). However, the federation is mainly focused on racing camels, and is specialized in implementing rules for fair racing, rather than defining breed standards.

The closest to “true” Arabian Peninsula camel breeds are the “Mezayen” camels, a term that literally means “beauty-contest” camels. The Mezayen camel breeds are the: Majaheem, Sofor, Shaele, Homor, Shageh, and Waddah (Abdallah and Faye, 2012; Porter et al., 2016; Alaibil Festival, 2017). We argue for their breed status because (1) each breed is defined by a distinct color group and a set recognized morphological features (Köhler-Rollefson, 1993), (2) a consensus of breed standards exists among breeders specifically for these six breeds (Teague, 2009), and because (3) an incentive to maintain breed standards is available in the form of camel beauty and breeding excellence competitions, such as the highly prized camel beauty competition of the King Abdulaziz Camel Festival (Alaibil Festival, 2017), along with more regional/tribal competitions (Hammond, 2007).

## Mezayen Phenotypes

The phenotypes and breed designations of domesticated animals are often more easily recognized by the breeder who selected for the particular traits. As such, we sought out Mezayen camel breeders to help in identifying and explaining the phenotypes of their camels that have been targets of selection using their common terminology. Mezayen camel breeders in the Arabian Peninsula use specific names to describe each breed (see above), breed subtype, and external phenotypes (Supplementary Tables S2, S3). The breed names and phenotypes described here are based on translations of the breeders’ Arabic terminology to ensure correct breed and phenotype assignments when collecting Cdrom Archive samples (see Supplementary Tables S2, S3 for details).

**FIGURE 4:**
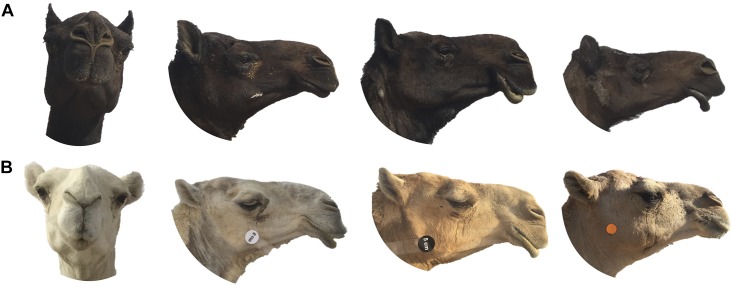
Ear length and shape variation between Mezayen camel breeds. **(A)** Majaheem camels have a distinct long-pointed ear shape, referred to as “speared” ears, whereas **(B)** Malaween breeds (Sofor, Shaele, Homor, Shageh, and Waddah) all exhibit shorter ears that are “folded” or “tilted” sideways and to the back. The white and black disks are five centimeters in diameter, which were added to extract a scale factor in subsequent morphometric analyses. Images were extracted from Cdrom Archive photos (collected by the authors).

**FIGURE 5:**
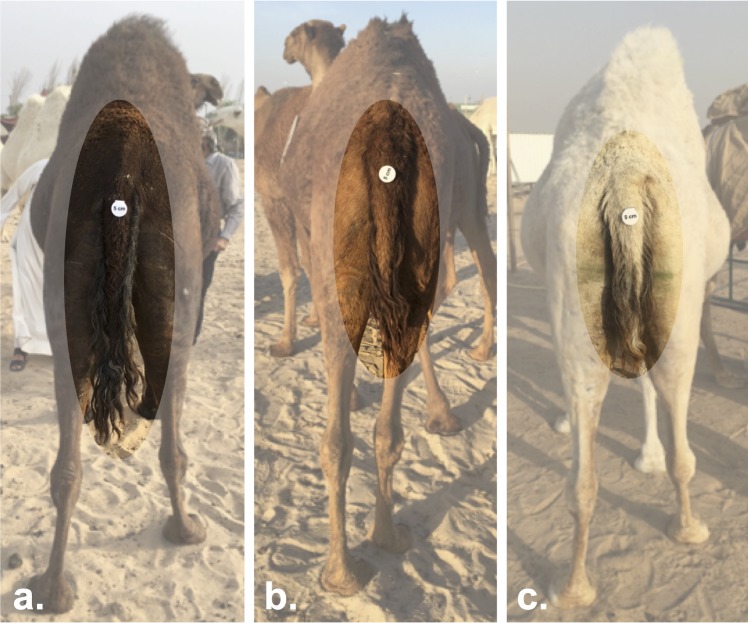
Tail length variation between Mezayen camel breeds. **(a)** Majaheem camels are characterized by long tails with a narrow tail-base. **(b)** Shaele, and **(c)** Waddah camels, which represent the Malaween breeds, display short-tails with wide tail-bases. The white disks on the tail are reference scales (five centimeters in diameter). Images were extracted from Cdrom Archive photos (collected by the authors).

Mezayen camels are divided into two main groups, the dark colored Majaheem, and the “Malaween,” which translates to colored breeds (Sofor, Shaele, Homor, Shageh, and Waddah) (personal observation). The separation of these two groups is in part based on coat color, but is also based on general features, such as body size, ear length and shape, and tail characteristics (Köhler-Rollefson, 1993; Abdallah and Faye, 2012). Majaheem camels are generally larger, and have long “speared” ears (Figure 4A), and a long tail with a narrow tail-base (Figure 5a) (Al-Hazmi et al., 1994). On the other hand, all Malaween breeds exhibit comparatively smaller body sizes, have short and tilted ears (Figure 4B), and a short tail with a wide tail-base (Figure 5b–c) (see Supplementary Table S2 for naming details). Breeders often do not breed Majaheem camels with any of the Malaween breeds, and when such an event occurs, breeders can easily recognize the hybrid due to changes in body features; such hybrids are often disqualified from competing in beauty competitions (personal observation). The Malaween are subdivided based on their coat color (Porter et al., 2016).

### Coat Color

**FIGURE 6:**
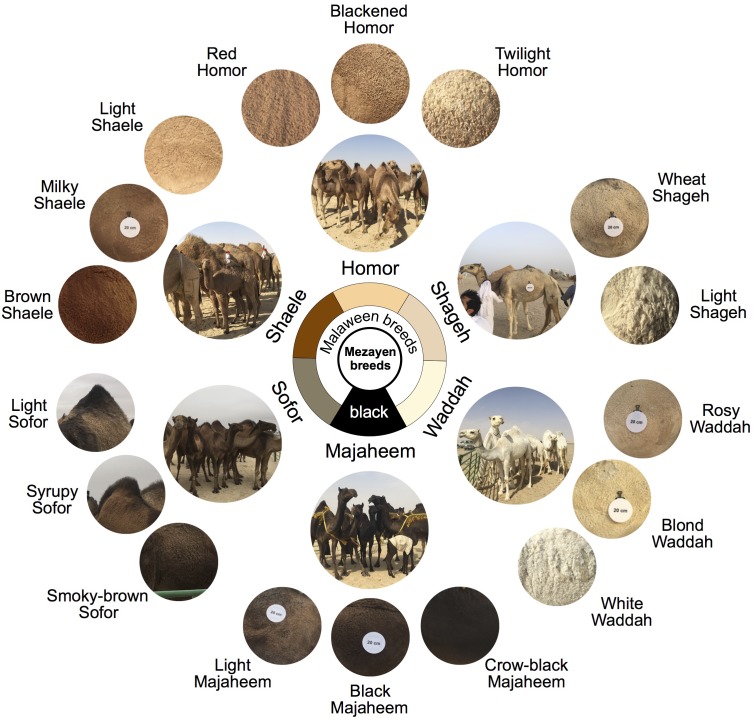
Mezayen camel breeds of the Arabia Peninsula and their coat colors. The six breeds are primarily divided into black vs. Malaween breeds. This division is based on coat color and morphological characteristics (e.g., ear shape and tail length). The black breed is Majaheem whereas the Malaween camels are further divided into five breeds based their coat color. The Malaween breeds, ranging from dark to light, are as follows: Sofor, Shaele, Homor, Shageh, and Waddah. The outer (small) circles represent the “sub-colors” of each breed. The Majaheem sub-colors are crow-black, black, and light. The Sofor sub-colors are smoky-brown, syrupy, and light. The Shaele sub-colors are brown, milky, and light. The Homor sub-colors are red, blackened, and twilight. The Shageh sub-colors are wheat and light. The Waddah sub-colors are rosy, blond, and white. Coat color circles come from the Cdrom Archive photographs and were extracted from the part of the lateral torso that is below the hump. The white disks are reference scales (20 centimeters in diameter). Breed photos were taken by Hasan Alhaddad during Mutair Cultural Festival 2017, Kuwait. Coat color circles were extracted from Cdrom Archive photos (collected by the authors).

Each Mezayen camel breed represents a color class (major color under which several varieties exist) (Figure 6). Broadly, the six color classes are black (Majaheem), smoky-brown (Sofor), brown (Shaele), red (Homor), wheat (Shageh), and white (Waddah) (Porter et al., 2016). Within each breed, a number of subtypes exist, which correspond to fine differences in coat color tone (Figure 6 – outer circle) (see Supplementary Table S2 for naming details). For example, under the broad black color class of the Majaheem, three subgroups are recognized. The sub-colors of Majaheem are (1) “crow-black” Majaheem, which as the name suggests, have a black coat color similar to the “blackness” of crow feathers, (2) “black” Majaheem are referred to by breeders as black, but is dark-brown color, that is similar to darkly roasted coffee beans, and (3) “light” Majaheem have a dark brown coat color with scattered light-colored hairs.

Camel coat colors have been recently investigated by sequencing two candidate genes *MC1R* (*melanocortin 1 receptor*) and *ASIP* (*agouti signaling protein*) (Almathen et al., 2018). Polymorphisms within the two candidate genes are found to be associated with broad color classifications (i.e., a single variant for black and dark brown colors) (Almathen et al., 2018). The color classifications presented here are more refined and are suspected to identify additional associated variants within *MC1R* and *ASIP* of each color (if they exist) or unravel a more complex genetic basis of coat color in camels.

### Hair Length

**FIGURE 7:**
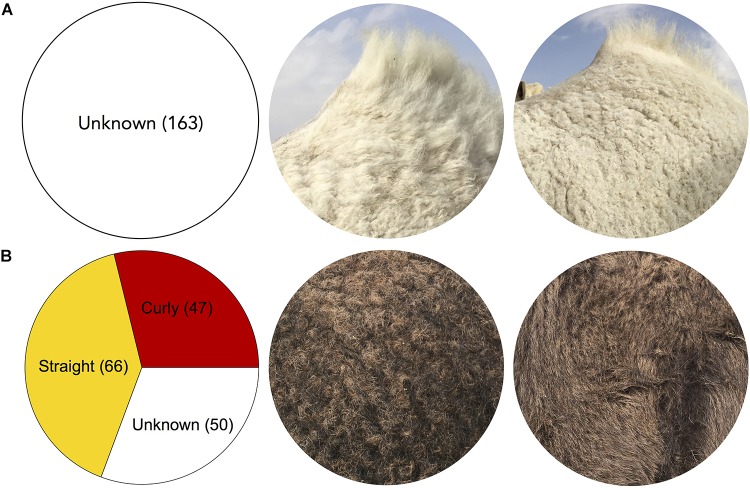
Camel coat length and texture variation. **(A)** Long and short coats, respectively. **(B)** “Ringed” (curly) and straight camel coat textures, respectively. The Cdrom Archive contains samples of both identified coat texture phenotypes from various Mezayen breeds **(B)**. Images were extracted from Cdrom Archive photos (collected by the authors).

Two hair length varieties (short and long) exist in each of the six Mezayen camels (Figure 7A). Breeders least favor the long-haired variety of each breed, especially when the hair texture is straight (personal observation). Thus, the identification of the molecular basis of hair length in camels may aid breeders in selecting camels to breed based on their genotype.

Fibroblast growth factor-5 (*FGF5*) has been identified to be associated with, or responsible for, long hair in many mammals, including llamas (Daverio et al., 2017), alpacas (Pallotti et al., 2018), donkeys (Legrand et al., 2014), sheep (Zhang et al., 2015), goats (Wang et al., 2016), cats (Drögemüller et al., 2007), dogs (Dierks et al., 2013), rabbits (Mulsant et al., 2010), mice (Hébert et al., 1994), and humans (Higgins et al., 2014). It is thus justifiable to consider *FGF5* as a strong candidate gene for long hair in Mezayen camels, and this hypothesis can be investigated via direct sequencing.

### Hair Texture

The hair texture of Mezayen camel coats comes into two varieties, straight and ringed (Figure 7B). These two varieties occur in all six breeds. Breeders select for curly hair that appear as rings, especially in the torso region, which are considered signs of beauty and health (personal observation). To achieve the most desirable coat for beauty competitions, breeders often select for a combination of short and ringed coat hairs (personal observation). The Crdom Archive currently contains 66 straight hair camels and 47 ringed hair camels, which we aim to use in genetic association studies—this relatively large sample size is optimal since a large number of genes are expected to be responsible for a curly coat (Figure 7B).

### “Syrupy” Sofor Coat Color

**FIGURE 8:**
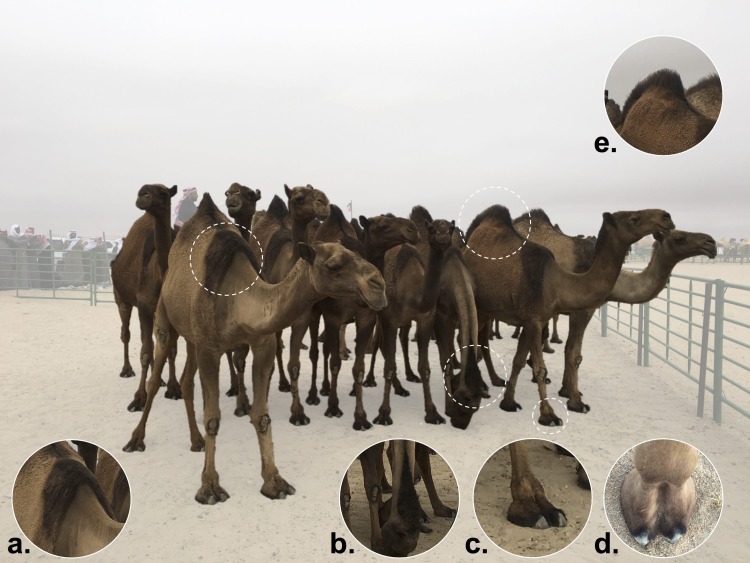
Dark coat “highlights” of the “Syrupy” Sofor phenotype. “Syrupy” Sofor camels show a darker coat color, located on **(a)** the withers, **(b)** the top of the neck, **(c,d)** the dorsal footpad and toes, and **(e)** the tip of the hump. The name of this breed is inspired by the camel breeders’ imagination, where the camel extremities “seem” as if they were “dipped in date syrup.” Photo was taken by Hasan Alhaddad during Mutair Cultural Festival 2017, Kuwait.

The “Syrupy” Sofor displays a unique coat color phenotype (see Supplementary Table S2 for naming details). This Sofor camel subtype shows a darker coat pigmentation at some body extremities, such as the withers, upper neck, dorsal footpad, nails, tip of the hump, and the tail (Figure 8). This phenotype does not occur in light colored breeds (Homor, Shagah, Waddah), but occasionally occurs in the Shaele breed, due to its intercrossing with the Sofor breed. This color phenotype has equivalents in other mammals, such as the “points” coat phenotype of Siamese and Burmese cats (Lyons et al., 2005), California rabbits (Aigner et al., 2000), and mice (Beermann et al., 2004).

Mutations in the *Tyrosinase* (*TRY*) gene have been associated with darker coloration in specific body parts, which arises due to the temperature sensitivity of gene production (Lyons et al., 2005). The close resemblance in coat phenotype between Syrupy Sofor camels, Siamese and Burmese cats, California rabbits, and mice, suggests that the *TYR* gene could be a strong candidate for this phenotype. Direct sequencing of the Syrupy Sofor camel genes, and the sequencing of the genes of their counterparts of the same breed (smoky-brown and light Sofor) could be a direct approach to study this phenotype.

## Camel Morphometrics

Several studies have examined the variation in body measurements among camel breeds (Al-Hazmi et al., 1994; Abdallah and Faye, 2012). So far, most published studies that investigate this theme use traditional, distance-based approaches, using calipers and measuring tape. While including such data along with each Cdrom Archive sample would provide valuable insights into the extent of the morphometric differentiation among the breeds, based on our personal experience, collecting such data manually is time-intensive and imprecise, given the temperamentality of most of the camels that we handled. Consequently, we developed a standardized method of photographing the sampled camels using the *SamplEase* application, where photographs are taken in such a way as to allow for the extraction of both linear and geometric morphometric data. We attach a scale bar to each sampled camel prior to photography to allow for the extraction of a scaling factor, which allows for the conversion of pixels to real units (i.e., centimeters). The “geometric morphometric” approach of examining morphological variation is commonly employed to extract data from zoological specimens (Zelditch et al., 2004; Alhajeri, 2018), and has recently been used to characterize morphological variation in live horses (Druml et al., 2015). More advanced methods of quantifying morphometric variation in camels in three-dimensions may also be implemented in the future.

## Conclusion

This review focused on outlining the framework of building and sample collection of our recently developed Cdrom Archive. This outline was intended to provide an example of how to establish a biobank that would be useful for genetic studies, thus we hope it would encourage others to establish similar camel biobanks elsewhere. Using the samples collected thus far, we introduced six camel breeds of the Arabian Peninsula that are used in camel beauty competitions and referred to as the Camel Mezayen contest. Using the photographs of the Cdrom archive, we discussed the coat color variations and their naming, as well as ear and tail variation. Where applicable, we outlined possible genetic approaches to study the genetics of these phenotypes and suggested likely candidate genes. Lastly, we introduced the possibility of applying morphometric tools to extract data from the photographs of the Cdrom Archive, which would allow us to investigate body size and shape variation. This review aimed to provide an example of what can be done across camel research laboratories to collect and characterize camel phenotypes, and possibly traits associated with production and adaptation for future genetic studies.

## Author Contributions

HA and BA collected the samples and wrote the manuscript.

## Conflict of Interest Statement

The authors declare that the research was conducted in the absence of any commercial or financial relationships that could be construed as a potential conflict of interest. The handling Editor and reviewer PO-tW declared their involvement as co-editors in the Research Topic, and confirm the absence of any other collaboration.

## Supplementary Material

TABLE S1 Camel terminology used to describe female camel age classes. Age terms were translated from original Arabic terms and the Arabic pronunciation is shown in italics. Similar names are given to male camels with slight differences related to gender changes to original terms in Arabic.

TABLE S2 Names of “Mezayen” camel breeds and their subtypes. Names of breeds and subtypes were translated from original Arabic terms and the Arabic pronunciation shown in italics. The translations are near exact to what is offered by the breeders, and in some cases the names do not exactly reflect the actual appearance. For visual comparison refer to Figure 6.

TABLE S3 Terminology of Mezayen camel coat texture and ear shape. Phenotype terminologies were translated from original Arabic terms and the Arabic pronunciation shown in italics.

TABLE S4 Current sample list and information of the Cdrom Archive.

## References

[B1] Abdallah H. R. Faye B. (2012). Phenotypic classification of Saudi Arabian camel (*Camelus dromedarius*) by their body measurements. *Emirates J. Food Agric.* 24 272–280.

[B2] Abu-seida A. Mostafa A. Tolba A. R. (2012). Anatomical and Ultrasonographical studies on tendons and digital cushions of normal phalangeal region in camels (*Camelus dromidarius*). *J. Camel Pract. Res.* 19 169–175.

[B3] Aigner B. Besenfelder U. Muller M. Brem G. (2000). Tyrosinase gene variants in different rabbit strains. *Mamm. Genome* 11 700–702. 10.1007/s003350010120 10920244

[B4] Alaibil Festival (2017). *King Abdulaziz Camel Festival.* Available: http://www.alaibilfestival.com/en/

[B5] Alhaddad H. Alhajeri B. H. (2018). SamplEase: a simple application for collection and organization of biological specimen data in the field. *Ecol. Evol.* 8 10266–10271. 10.1002/ece3.4503 30397464 PMC6206197

[B6] Alhaddad H. Maraqa T. Alabdulghafour S. Alaskar H. Alaqeely R. Almathen F. (2019). Quality and quantity of dromedary camel DNA sampled from whole-blood, saliva, and tail-hair. *PLoS One* 14:e0211743 10.1371/journal.pone.0211743 PMC6355012 30703133

[B7] Alhajeri B. H. (2018). Cranial variation in geographically widespread dwarf gerbil *Gerbillus nanus* (Gerbillinae, Rodentia) populations: isolation by distance versus adaptation to local environments. *J. Zool. Syst. Evol. Res.* 9 928–937. 10.1111/jzs.12247

[B8] Al-Hazmi M. Ghandour A. Elgohar M. (1994). A study of the biometry of some breeds of arabian camel (came/us dromedarius) in Saudi Arabia. *J. King Abdulaziz Univ.* 6 87–99. 10.4197/Sci.6-1.7

[B9] Ali A. Derar D. Alsharari A. Alsharari A. Khalil R. Almundarij T. I. (2018). Factors affecting reproductive performance in dromedary camel herds in Saudi Arabia. *Trop. Anim. Health Prod.* 50 1155–1160. 10.1007/s11250-018-1545-3 29450815

[B10] Almathen F. Elbir H. Bahbahani H. Mwacharo J. Hanotte O. (2018). Polymorphisms in MC1R and ASIP genes are associated with coat colour variation in the Arabian camel. *J. Hered.* 109 700–706. 10.1093/jhered/esy024 29893870 PMC6108395

[B11] Al-Swailem A. M. Shehata M. M. Abu-Duhier F. M. Al-Yamani E. J. Al-Busadah K. A. Al-Arawi M. S. (2010). Sequencing, analysis, and annotation of expressed sequence tags for *Camelus dromedarius*. *PLoS One* 5:e10720. 10.1371/journal.pone.0010720 20502665 PMC2873428

[B12] Beermann F. Orlow S. J. Lamoreux M. L. (2004). The Tyr (albino) locus of the laboratory mouse. *Mamm. Genome* 15 749–758. 10.1007/s00335-004-4002-8 15520878

[B13] Blackburn H. D. (2018). Biobanking genetic material for agricultural animal species. *Annu. Rev. Anim. Biosci.* 6 69–82. 10.1146/annurev-animal-030117-014603 29220198

[B14] Burger P. A. (2016). The history of Old World camelids in the light of molecular genetics. *Trop. Anim. Health Prod.* 48 905–913. 10.1007/s11250-016-1032-7 27048619 PMC4884201

[B15] Cho I.-C. Zhong T. Seo B.-Y. Jung E.-J. Yoo C.-K. Kim J.-H. (2011). Whole-genome association study for the roan coat color in an intercrossed pig population between Landrace and Korean native pig. *Genes Genomics* 33 17–23. 10.1007/s13258-010-0108-4

[B16] Daverio M. S. Vidal-Rioja L. Frank E. N. Di Rocco F. (2017). Molecular characterization of the llama FGF5 gene and identification of putative loss of function mutations. *Anim. Genet.* 48 716–719. 10.1111/age.12616 29024003

[B17] David V. A. Menotti-Raymond M. Wallace A. C. Roelke M. Kehler J. Leighty R. (2014). Endogenous retrovirus insertion in the KIT oncogene determines white and white spotting in domestic cats. *G3* 4 1881–1891. 10.1534/g3.114.013425 25085922 PMC4199695

[B18] Dierks C. Momke S. Philipp U. Distl O. (2013). Allelic heterogeneity of FGF5 mutations causes the long-hair phenotype in dogs. *Anim. Genet.* 44 425–431. 10.1111/age.12010 23384345

[B19] Dioli M. (2016). Towards a rational camel breed judging: a proposed standard of a camel (*Camelus dromedarius*) milk breed. *J. Camel Pract. Res.* 23 1–12. 10.5958/2277-8934.2016.00001.1

[B20] Drögemüller C. Rüfenacht S. Wichert B. Leeb T. (2007). Mutations within the FGF5 gene are associated with hair length in cats. *Anim. Genet.* 38 218–221. 10.1111/j.1365-2052.2007.01590.x 17433015

[B21] Druml T. Dobretsberger M. Brem G. (2015). The use of novel phenotyping methods for validation of equine conformation scoring results. *Animal* 9 928–937. 10.1017/S1751731114003309 25582051

[B22] Eshra E. A. Badawy A. M. (2014). Peculiarities of the camel and sheep narial musculature in relation to the clinical value and the mechanism of narial closure. *Indian J. Vet. Anat.* 26 10–13.

[B23] Farah Z. (1993). Composition and characteristics of camel milk. *J. Dairy Res.* 60 603–626. 10.1017/S0022029900027953 8294611

[B24] Fitak R. R. Mohandesan E. Corander J. Burger P. A. (2016). The de novo genome assembly and annotation of a female domestic dromedary of North African origin. *Mol. Ecol. Resour.* 16 314–324. 10.1111/1755-0998.12443 26178449 PMC4973839

[B25] Fontanesi L. Tazzoli M. Russo V. Beever J. (2010). Genetic heterogeneity at the bovine KIT gene in cattle breeds carrying different putative alleles at the spotting locus. *Anim. Genet.* 41 295–303. 10.1111/j.1365-2052.2009.02007.x 19968642

[B26] Fontanesi L. Vargiolu M. Scotti E. Latorre R. Faussone Pellegrini M. S. Mazzoni M. (2014). The KIT gene is associated with the english spotting coat color locus and congenital megacolon in checkered giant rabbits (*Oryctolagus cuniculus*). *PLoS One* 9:e93750. 10.1371/journal.pone.0093750 24736498 PMC3988019

[B27] Food Agriculture Organization of the United Nations (2013). *In Vivo Conservation of Animal Genetic Resources.* Rome: Food and Agriculture Organization of the United Nations.

[B28] Geissler E. N. Ryan M. A. Housman D. E. (1988). The dominant-white spotting (W) locus of the mouse encodes the *c-kit* proto-oncogene. *Cell* 55 185–192. 10.1016/0092-8674(88)90020-7 2458842

[B29] Groeneveld L. F. Gregusson S. Guldbrandtsen B. Hiemstra S. J. Hveem K. Kantanen J. (2016). Domesticated animal biobanking: land of opportunity. *PLoS Biol.* 14:e1002523. 10.1371/journal.pbio.1002523 27467395 PMC4965055

[B30] Haase B. Rieder S. Leeb T. (2015). Two variants in the KIT gene as candidate causative mutations for a dominant white and a white spotting phenotype in the donkey. *Anim. Genet.* 46 321–324. 10.1111/age.12282 25818843

[B31] Hammond A. (2007). *Saudi tribe holds camel beauty pageant. Oddly Enough*. Available at: https://www.reuters.com/article/us-saudi-camels-beauty-odd/saudi-tribe-holds-camel-beauty-pageant-idUSKUA74812720070427

[B32] Hauswirth R. Jude R. Haase B. Bellone R. R. Archer S. Holl H. (2013). Novel variants in the KIT and PAX3 genes in horses with white-spotted coat colour phenotypes. *Anim. Genet.* 44 763–765. 10.1111/age.12057 23659293

[B33] Hébert J. M. Rosenquist T. Götz J. Martin G. R. (1994). FGF5 as a regulator of the hair growth cycle: evidence from targeted and spontaneous mutations. *Cell* 78 1017–1025. 10.1016/0092-8674(94)90276-3 7923352

[B34] Higgins C. A. Petukhova L. Harel S. Ho Y. Y. Drill E. Shapiro L. (2014). FGF5 is a crucial regulator of hair length in humans. *Proc. Natl. Acad. Sci. U.S.A.* 111 10648–10653. 10.1073/pnas.1402862111 24989505 PMC4115575

[B35] Hirschhorn J. N. Daly M. J. (2005). Genome-wide association studies for common diseases and complex traits. *Nat. Rev. Genet.* 6 95–108. 10.1038/nrg1521 15716906

[B36] Holl H. Isaza R. Mohamoud Y. Ahmed A. Almathen F. Youcef C. (2017). A frameshift mutation in KIT is associated with white spotting in the arabian camel. *Genes* 8:E102. 10.3390/genes8030102 28282952 PMC5368706

[B37] Jackling F. C. Johnson W. E. Appleton B. R. (2014). The genetic inheritance of the blue-eyed white phenotype in alpacas (*Vicugna pacos*). *J. Hered.* 105 847–857. 10.1093/jhered/ess093 23144493 PMC4201308

[B38] Kadim I. T. Mahgoub O. Purchas R. W. (2008). A review of the growth, and of the carcass and meat quality characteristics of the one-humped camel (*Camelus dromedaries*). *Meat. Sci.* 80 555–569. 10.1016/j.meatsci.2008.02.010 22063567

[B39] Kagunyu A. W. Matiri F. Ngari E. (2013). Camel hides: production, marketing and utilization in pastoral regions of northern Kenya. *Pastoralism* 3:25 10.1186/2041-7136-3-25

[B40] Kahle D. Wickham H. (2013). ggmap: spatial visualization with ggplot2. *R J.* 5 144–161.

[B41] Karlsson E. K. Lindblad-Toh K. (2008). Leader of the pack: gene mapping in dogs and other model organisms. *Nat. Rev. Genet.* 9:713. 10.1038/nrg2382 18714291

[B42] Khalaf S. (1999). Camel racing in the gulf. Notes on the evolution of a traditional cultural sport. *Anthropos* 94 85–106.

[B43] Köhler-Rollefson I. (1993). About camel breeds: a reevaluation of current classification systems. *J. Anim. Breed. Genet.* 110 66–73. 10.1111/j.1439-0388.1993.tb00717.x 21395704

[B44] Legrand R. Tiret L. Abitbol M. (2014). Two recessive mutations in FGF5 are associated with the long-hair phenotype in donkeys. *Genet. Sel. Evol.* 46:65. 10.1186/s12711-014-0065-5 25927731 PMC4175617

[B45] Lloyd-Jones O. (1915). WHAT IS A BREED? Definition of word varies with each kind of livestock, and is based almost wholly on arbitrary decision of breeders—some strange contradictions—the meaning of “Pure-Bred”1. *J. Hered.* 6 531–537. 10.1093/oxfordjournals.jhered.a109038

[B46] Lyons L. A. Imes D. L. Rah H. C. Grahn R. A. (2005). Tyrosinase mutations associated with Siamese and Burmese patterns in the domestic cat (*Felis catus*). *Anim. Genet.* 36 119–126. 10.1111/j.1365-2052.2005.01253.x 15771720

[B47] Mulsant P. De Rochambeau H. Thébault R. G. (2010). A note on linkage between the Angora and fgf5 genes in rabbits. *World Rabbit Sci.* 12 1–6. 10.4995/wrs.2004.585

[B48] Nazari-Ghadikolaei A. Mehrabani-Yeganeh H. Miarei-Aashtiani S. R. Staiger E. A. Rashidi A. Huson H. J. (2018). Genome-wide association studies identify candidate genes for coat color and mohair traits in the iranian markhoz goat. *Front. Genet.* 9:105. 10.3389/fgene.2018.00105 29670642 PMC5893768

[B49] Omrani A. S. Al-Tawfiq J. A. Memish Z. A. (2015). Middle East respiratory syndrome coronavirus (MERS-CoV): animal to human interaction. *Pathog. Glob. Health* 109 354–362. 10.1080/20477724.2015.1122852 26924345 PMC4809235

[B50] Ott J. Wang J. Leal S. M. (2015). Genetic linkage analysis in the age of whole-genome sequencing. *Nat. Rev. Genet.* 16 275–284. 10.1038/nrg3908 25824869 PMC4440411

[B51] Pallotti S. Pediconi D. Subramanian D. Molina M. G. Antonini M. Morelli M. B. (2018). Evidence of post-transcriptional readthrough regulation in FGF5 gene of alpaca. *Gene* 647 121–128. 10.1016/j.gene.2018.01.006 29307854

[B52] Petersen B.-S. Fredrich B. Hoeppner M. P. Ellinghaus D. Franke A. (2017). Opportunities and challenges of whole-genome and -exome sequencing. *BMC Genetics* 18:14. 10.1186/s12863-017-0479-5 28193154 PMC5307692

[B53] Porter V. Alderson L. Hall S. J. G. Sponenberg D. P. (2016). *Mason’s World Encyclopedia of Livestock Breeds and Breeding.* Boston, MA: CABI, 1 10.1079/9781845934668.0000

[B54] R Development Core Team (2018). *R: A Language and Environment for Statistical Computing.* Vienna: R Foundation for Statistical Computing.

[B55] Ramadan S. Nowier A. M. Hori Y. Inoue-Murayama M. (2018). The association between glutamine repeats in the androgen receptor gene and personality traits in dromedary camel (*Camelus dromedarius*). *PLoS One* 13:e0191119. 10.1371/journal.pone.0191119 29415053 PMC5802489

[B56] Schmidt-Nielsen K. (1959). The physiology of the camel. *Sci. Am.* 201 140–151. 10.1038/scientificamerican1259-140 14443122

[B57] Teague M. (2009). *Isn’t She Lovely.* Washington, DC: National Geographic.

[B58] Volpato G. Dioli M. Di Nardo A. (2017). Piebald camels. *Pastoralism* 7:3 10.1186/s13570-017-0075-3 PMC7114962 32269746

[B59] Wang X. Cai B. Zhou J. Zhu H. Niu Y. Ma B. (2016). Disruption of FGF5 in cashmere goats using CRISPR/Cas9 results in more secondary hair follicles and longer fibers. *PLoS One* 11:e0164640. 10.1371/journal.pone.0164640 27755602 PMC5068700

[B60] Wardeh M. F. (2004). Classification of the dromedary camels. *J. Camel Sci.* 1 1–7.

[B61] Wong A. K. Ruhe A. L. Robertson K. R. Loew E. R. Williams D. C. Neff M. W. (2012). A de novo mutation in KIT causes white spotting in a subpopulation of German Shepherd dogs. *Anim. Genet.* 44 305–310. 10.1111/age.12006 23134432

[B62] Wu H. Guang X. Al-Fageeh M. B. Cao J. Pan S. Zhou H. (2014). Camelid genomes reveal evolution and adaptation to desert environments. *Nat. Commun.* 5:5188. 10.1038/ncomms6188 25333821

[B63] Zelditch M. L. Swiderski D. L. Sheets H. D. Fink W. L. (2004). *Geometric Morphometrics for Biologists.* San Diego, CA: Academic Press.

[B64] Zhang L. He S. Liu M. Liu G. Yuan Z. Liu C. (2015). Molecular cloning, characterization, and expression of sheep FGF5 gene. *Gene* 555 95–100. 10.1016/j.gene.2014.10.036 25445274

[B65] Zhang M. Q. Xu X. Luo S. J. (2014). The genetics of brown coat color and white spotting in domestic yaks (Bos grunniens). *Anim. Genet.* 45 652–659. 10.1111/age.12191 24989079

[B66] Zhu M. Zhao S. (2007). Candidate gene identification approach: progress and challenges. *Int. J. Biol. Sci.* 3 420–427. 10.7150/ijbs.3.420 17998950 PMC2043166

