# Supplementary material for: Cdrom Archive: A Gateway to Study Camel Phenotypes
Source: Front Genet. 2019 Feb 5;10:48. doi: 10.3389/fgene.2019.00048 (PMC6370635; doi:10.3389/fgene.2019.00048)
Supplement: TABLE S1 — Camel terminology used to describe female camel age classes. Age terms were translated from original Arabic terms and the Arabic pronunciation is shown in italics. Similar names are given to male camels with slight differences related to gender changes to original terms in Arabic. [file Table_1.DOCX]

**Supplementary Table 1: Camel terminology used to describe female camel age classes.** Age terms were translated from original Arabic terms and the Arabic pronunciation is shown in italics. Similar names are given to male camels with slight differences related to gender changes to original terms in Arabic.

| **Age** **Class**  Original Arabic  (*Pronunciation*) | **Description** | **Approximate Age**  **(Years)** |
| --- | --- | --- |
| **The Bewildered**  حواره  (*Howarah*) | The age class name is given to the female camel from its birth and throughout its weaning period. The term is given because the newly born camel seems bewildered and seeking the mother’s protection and care. This stage ends when the camel no longer relies on milk as a primary source of nutrition. | 0-1 |
| **The Solitary**  مفروده  (*Mafroudah*) | The age class name is given to the female camel when it is independent from the mother and can feed on its own. Note that at this stage the mother can become pregnant again. | 1-2 |
| **The Encountered**  لقيَّه  (*Leqiyah*) | The age class name is given to the female camel when it encounters its newly born sibling, hence the name. | 2-3 |
| **The Eligible**  حِقَّه  (*Heqah*) | The age class name is given to the female camel that is physically able to be ridden, carry loads, or mate. | 3-4 |
| **The Young-adult**  جذعه  (*Jethaa*) | The age class name is given to the female camel that is reproductively mature and can be mated. Generally, this is the age class when camel breeders in the Arabian Peninsula start breeding the female. | 4-5 |
| **The Doubled**  ثنيِّه  (*Theniyah*) | The age class name is given to the female camel when the first pair of permanent front incisors of the lower jaw appears. | 5-6 |
| **The Quadrupled**  رباع  (*Rubaa*) | The age class name is given to the female camel when the second pair of permanent front incisors of the lower jaw appears (total front incisors are now four). | 6-7 |
| **The Sextupled**  سديس  (*Sidees*) | The age class name is given to the female camel when the third pair of permanent front incisors of the lower jaw appears (total front incisors are now six). At this stage the camel has a full set of permanent front lower jaw teeth. | 7-8 |
| **Naqah**  ناقه  (*Naqah*) | A general name given to any female camel that is reproductively mature. This term is used from after the first mating and until death. | > 4 |
| **The Erupted**  فاطر أو شاق  (*Fater or Shag*) | The age class name is given to the female camel when the canine teeth appears. The term may also be used to refer to old female camels. | > 8 |
| **The Virgin**  بكره  (*Bakrah*) | A general name given to the female camel that is reproductively immature. | 1-3 |
