# Supplementary material for: Cdrom Archive: A Gateway to Study Camel Phenotypes
Source: Front Genet. 2019 Feb 5;10:48. doi: 10.3389/fgene.2019.00048 (PMC6370635; doi:10.3389/fgene.2019.00048)
Supplement: TABLE S2 — Names of “Mezayen” camel breeds and their subtypes. Names of breeds and subtypes were translated from original Arabic terms and the Arabic pronunciation shown in italics. The translations are near exact to what is offered by the breeders, and in some cases the names do not exactly reflect the actual appearance. For visual comparison refer to Figure 6. [file Table_2.DOCX]

**Supplementary Table 2: Names of ‘Mezayen' camel breeds and their subtypes.** Names of breeds and subtypes were translated from original Arabic terms and the Arabic pronunciation shown in italics. The translations are near exact to what is offered by the breeders, and in some cases the names do not exactly reflect the actual appearance. For visual comparison refer to Figure 6.

| **Name**  Original Arabic  (*Pronunciation*) | **Description** |
| --- | --- |
| **Mezayen**  مزاين  (*Mezayen*) | A term that literally means “beauty contest”. It is generally used for camel beauty and breeding excellence competitions. |
| **Malaween**  ملاوين  (*Malaween*) | A general term given to all colored Mezayen (camel beauty contest) breeds (Sofor, Shaele, Homor, Shageh, and Waddah) excluding the dark colored Majaheem. While colored breeds differ in coat color, they all share the characteristics of short and tilted ears, short tail, and wide tail base. |
| **Majaheem**  مجاهيم  (*Mejaheem*) | A Mezayen camel breed that is recognized for its dark brown-to-black color. It is also characterized by a large body, long speared ears (vertical and pointed), long tail, and a narrow tail base. This breed produces a high volume of milk. Majaheem is a plural term—the singular term is Mejhem for a male or Mejehma for a female. |
| **‘Crow-black’ Majaheem**  مجاهيم سوداء احسنيّه أو غرابيه  (*Souda Ehseniya or Ghurabiya*) | A subtype of Majaheem camels recognized by its black color. The black color of the coat is similar to the black feathers of a crow, and hence the name. |
| **Black Majaheem**  مجاهيم سوداء  (*Mejaheem Souda*) | A subtype of Majaheem camels that is recognized by its dark brown color. The dark brown color of the coat is similar to a very dark roast of coffee beans. Note that the name is not an exact reflection of the coat color but it is what breeders use. |
| **Light Majeheem**  مجاهيم صهباء  (*Mejaheem Sahbaa*) | A subtype of Mejaheem camels that is recognized by its dark brown color with hints of lighter hairs. |
| **Sofor**  صفر  (*Sofor*) | A Mezayen camel breed that is recognized for its general smoky-brown coat color. It is also known for short tilted ears (back pointing), short tail, and a wide tail base. Sofor is a plural term—the singular term is Asfar (male) and Safra (female). |
| **Smoky-brown Sofor**  صفر دلماء  (*Sofor Dalmaa*) | A subtype of Sofor camels, recognized by a uniform smoky-brown coat color across all body parts. |
| **‘Syrupy’ Sofor**  صفر دباسيه  (*Sofor Dubasiya*) | A subtype of Sofor camels recognized by a uniform smoky-brown coat color across all body parts, in addition to a darker coat at the tip of the hump, tail, foot (nail base), and the upper parts of the neck. The name translates to “date syrup” as if the darker extremities are dipped in syrup. |
| **Light Sofor**  صفر شهباء  (*Sofor Shahbaa*) | A subtype of Sofor camels recognized by a uniform smoky-brown coat color across all body parts, with small white hairs. |
| **Shaele**  شعل  (*Shuel*) | A Mezayen camel breed recognized by its uniform brown coat color across all body parts. It is also characterized by short tilted ears (pointing backwards), short tail, and a wide tail base. Sahele is a plural term—the singular term is Ashale (male) or Eshala (female). |
| **Brown Shaele**  شعل دعماء  (*Shuel Damaa*) | A subtype of Shaele camels recognized by a uniform brown coat color across all body parts. |
| **Milky Shaele**  شعل حبشاء  (*Shuel Habshaa*) | A subtype of Shaele camels recognized by a uniform milky brown coat color across all body parts. |
| **Light Shaele**  شعل بياضيه  (*Shuel Beyadiya*) | A subtype of Shaele camels recognized by a uniform light brown coat color across all body parts. |
| **Homor**  حمر  (*Humor*) | A Mezayen camel breed recognized for its red coat color. It is also characterized by short tilted ears (pointing backwards), short tail, and a wide tail base. Homor is a plural term—the singular term is Ahmar (male) or Hamra (female). |
| **‘Twilight’ Homor**  حمر شفقاء  (*Humor Shafqaa*) | A subtype of Homor camels recognized by ‘twilight’ colors, where lower body parts are creamy colored and upper body parts are pinkish red. |
| **Blackened Homor**  حمر دهماء  (*Humor Dahmaa*) | A subtype of Homor camels recognized by a pinkish-red coat color across all body parts, with small scattered black hairs, especially on top of the withers. |
| **Red Homor**  حمر مِجوِخ  (*Humor Mijwekh*) | A subtype of Homor camels recognized by a uniform pinkish-red coat color across all body parts and an absence of any dark colored hairs. |
| **Shageh**  شقح  (*Shegeh*) | A Mezayen camel breed recognized by its cream coat color, with dark hair ‘impurities’ (similar to the color of wheat). It is also known for short tilted ears (pointing backwards), short tail, and a wide tail base. Shageh is a plural term—the singular is Ashagah (male) or Shageha (female). |
| **Wheat Shageh**  شقح قمحية  (*Shegeh Qamhiyah*) | A subtype of Sahgeh camels recognized by a uniform wheat coat color (creamy color with scattered dark hairs) across all body parts. |
| **Light Shageh**  شقح بياضية  (*Shegeh Beyadiya*) | A subtype of Sahgeh camels recognized by a uniform light wheat coat color (creamy color with scattered lighter hairs) across all body parts. |
| **Waddah**  وضح  (*Wodeh*) | A Mezayen camel breed recognized by its white creamy coat color. It is also characterized by short tilted ears (pointing backwards), short tail, and a wide tail base. Waddah is a plural term—the singular term is Awdah (male) or Wadeha (female). |
| **Rosy Waddah**  بشه، عطره، دغماء  (*Basha, Atraa, Daghmaa*) | A subtype of Waddah camels recognized by its creamy white color, with some light rosy-pink colored hairs. |
| **Blonde Waddah**  وضح شقراء  (*Wodeh Shaqraa*) | A subtype of Waddah camels recognized by its creamy-white color, with some golden blonde hairs. |
| **White Waddah**  وضح بيضاء  (*Wodeh Baidhah*) | A subtype of Waddah camels recognized by its uniform creamy-white color across all body parts. This subtype can be further divided into eye-lined or albino Waddah. |
| **Eye-lined Waddah**  وضح مكحله، دعجاء  (*Wodeh Mukahala, Dajaa*) | A subtype of Waddah camels that is similar to the white Waddah but has distinct black hair coloration in the eye and lips (the facial features are outlined by black hairs). |
| **Albino Waddah**  بلقاء، برصاء  (*Balqaa, Barsaa*) | A rare subtype of Waddah camels that is white colored on all parts of the body including the eyelids and mouth. |
