# Supplementary material for: Cdrom Archive: A Gateway to Study Camel Phenotypes
Source: Front Genet. 2019 Feb 5;10:48. doi: 10.3389/fgene.2019.00048 (PMC6370635; doi:10.3389/fgene.2019.00048)
Supplement: TABLE S3 — Terminology of Mezayen camel coat texture and ear shape. Phenotype terminologies were translated from original Arabic terms and the Arabic pronunciation shown in italics. [file Table_3.DOCX]

**Supplementary** **Table 3: Terminology of Mezayen camel coat texture and ear shape.** Phenotype terminologies were translated from original Arabic terms and the Arabic pronunciation shown in italics.

| **Term**  Original Arabic  (*Pronunciation*) | **Description** | **Reference Figure** |
| --- | --- | --- |
| **Straight coat**  وبرة طلقاء أو ملساء  (*Wabra Talqa, Malsaa*) | A variation of coat texture that is found in all Mezayen breeds and least favored by camel breeders. | 7b |
| **Ringed coat**  وبرة مِحْلِقْ، معكرشه  (*Wabra Mehleq, Muakresha*) | A curly coat texture that is found in all Mezayen camel breeds and favored by breeders. In a “ringed” coat, the hair curls form a circular ring shape. | 7b |
| **Speared ears**  حداد، حراب  (*Hedad, Herab*) | Ear shape unique to Mejaheem camels, which is straight and pointed upwards. | 4a |
| **Tilted ears**  خرع  (*Kheraa*) | Ear shape unique to the colored Mezayen camel breeds, which is tilted backwards. | 4b |
